# A far-field radio-frequency experimental exposure system with unrestrained mice

**DOI:** 10.1186/s40064-015-1433-5

**Published:** 2015-11-04

**Authors:** Jared W. Hansen, Sajid Asif, Lauren Singelmann, Muhammad Saeed Khan, Sumit Ghosh, Tom Gustad, Curt Doetkott, Benjamin D. Braaten, Daniel L. Ewert

**Affiliations:** Department of Electrical and Computer Engineering, North Dakota State University, NDSU Dept 2480, PO Box 6050, Fargo, ND 58108-6050 USA; Department of Veterinary and Microbiological Sciences, North Dakota State University, Fargo, ND 58102 USA; Statistics Consulting Services, North Dakota State University, Fargo, ND 58102 USA; Department of Information Engineering, University of Padova, 35131 Padua, Italy; Department of Electrical Engineering, COMSATS Institution of Information Technology, Attock, Pakistan

**Keywords:** In vivo, Microstrip, Radio-frequency, Specific absorption rate

## Abstract

Many studies have been performed on exploring the effects of radio-frequency (RF) energy on biological function in vivo. In particular, gene expression results have been inconclusive due, in part, to a lack of a standardized experimental procedure. This research describes a new far field RF exposure system for unrestrained murine models that reduces experimental error. The experimental procedure includes the materials used, the creation of a patch antenna, the uncertainty analysis of the equipment, characterization of the test room, experimental equipment used and setup, power density and specific absorption rate experiment, and discussion. The result of this research is an experimental exposure system to be applied to future biological studies.

## Background

Radio-frequency (RF) energy is nearly everywhere, it is used in cell phones, wireless internet and many other sources. These RF energy levels used by common devices are below the threshold level which does not produce heating of cells in living tissues. However, this low-level exposure of RF energy has still raised concerns over its possible effects on human health, specifically, genetic alterations. Researchers have investigated if RF energy can induce changes in biological function (Gherardini 2014; Kundi 2009; Polk and Postow 1995; Vanderstaeten and Verschaeve 2008). The methods used to investigate RF energy effects have varied widely depending on study. This variation in procedures has led to a lack of reproducibility, and because of that, inconclusive results (Gherardini 2014; Vanderstaeten and Verschaeve 2008). The goal of this paper is to describe a new experimental exposure system to explore the effects of far-field RF energy on biological function in unrestrained murine models, in vivo.

Paffi et al. (2013) performed an extensive review of exposure systems. Many of these used a horn antenna to deliver RF energy, but lack long term continuous exposure for free moving murine models. Other studies including Kesari et al. (2010) and Wasoontarajaroen et al. (2012) used intermittent RF exposure, and still others in Paffi et al. (2013) used a reverberation chamber to deliver RF energy. For this work, an RF amplifier, horn antenna and anechoic chamber material were used to; (1) provide a more precisely defined RF field for accurate long-term exposure in freely moving test subjects and (2) the uncertainty analysis is more convenient as opposed to a reverberation chamber. This exposure system coupled with uncertainty analysis addresses many of the shortcomings stated in Workshop on EMF and Health Risk Research 2012 Monte Verità, Switzerland.

This paper describes the equipment and materials used, creation of a patch antenna, uncertainty analysis of the equipment, characterization of the background RF energy in the test room, specific absorption rate calculation, and setup of the equipment used for testing far-field RF exposure on unrestrained murine models.

## Methods

**Fig. 1:**
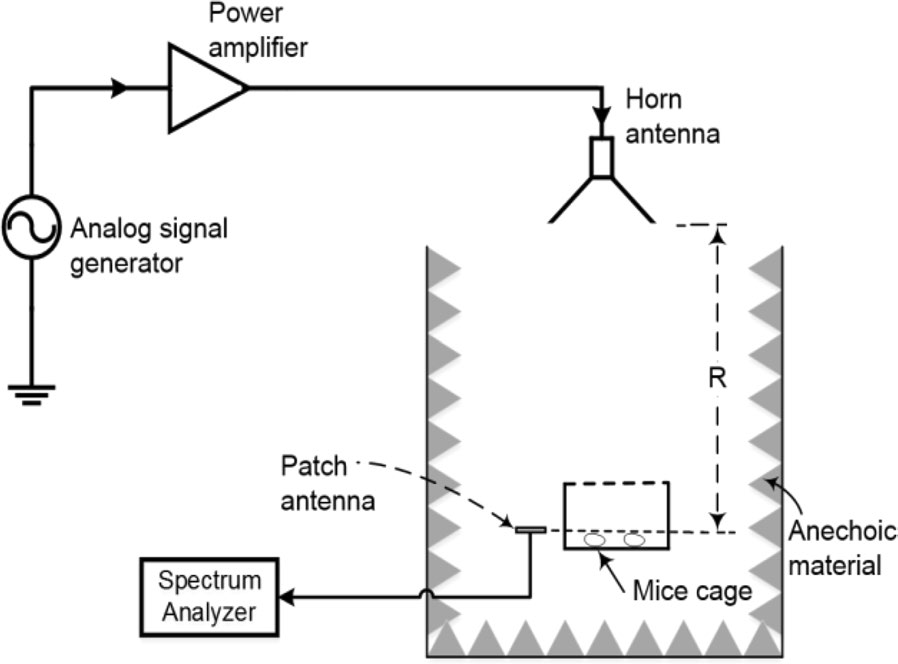
Layout of the experimental setup for RF exposure of mice with all the apparatus used. The transmitter antenna (*Horn*) is at the distance R from the mice cage

Figure 1 depicts the experimental setup for the RF exposure system for unrestrained murine models, in vivo. The figure shows a horn antenna connected to a power amplifier and signal generator, placed a distance ‘R’ away from the mice cage. The levels of RF are measured by a patch antenna connected to a spectrum analyzer. Table 1 lists the equipment used, and the make/model/specifications.

**Table 1:** Equipment used

S. no.	Name	Make/model/specifications
1.	Analog signal generator	Agilent/N5181A 100 kHz–3 GHz
2.	Spectrum analyzer	Agilent E4402B 9 kHz–3 GHz
3.	Horn antenna	TDK RF Solutions (HRN-0118) 1–18 GHz
4.	Patch antenna	Manufactured on TMM4 f = 2.43 GHz, gain = 4.8 dBi thickness = 1.52 mm and 0.5 oz. copper
5.	Biconical antenna	A.H. Systems (SAS-521-4) 25 MHz–4 GHz
6.	Coax cable	UTIFLEX Micro-Coax 26.5 GHz
7.	Power amplifier	Mini-circuits (ZHL-30W-252-S+) 700–2500 MHz
8.	Mouse cage	Plastic (20 × 30 × 16 cm^3^)
9.	Anechoic material	MVG AEP-18 (pyramid absorber) 30 MHz–18 GHz

### Uncertainty analysis of equipment

**Fig. 2:**
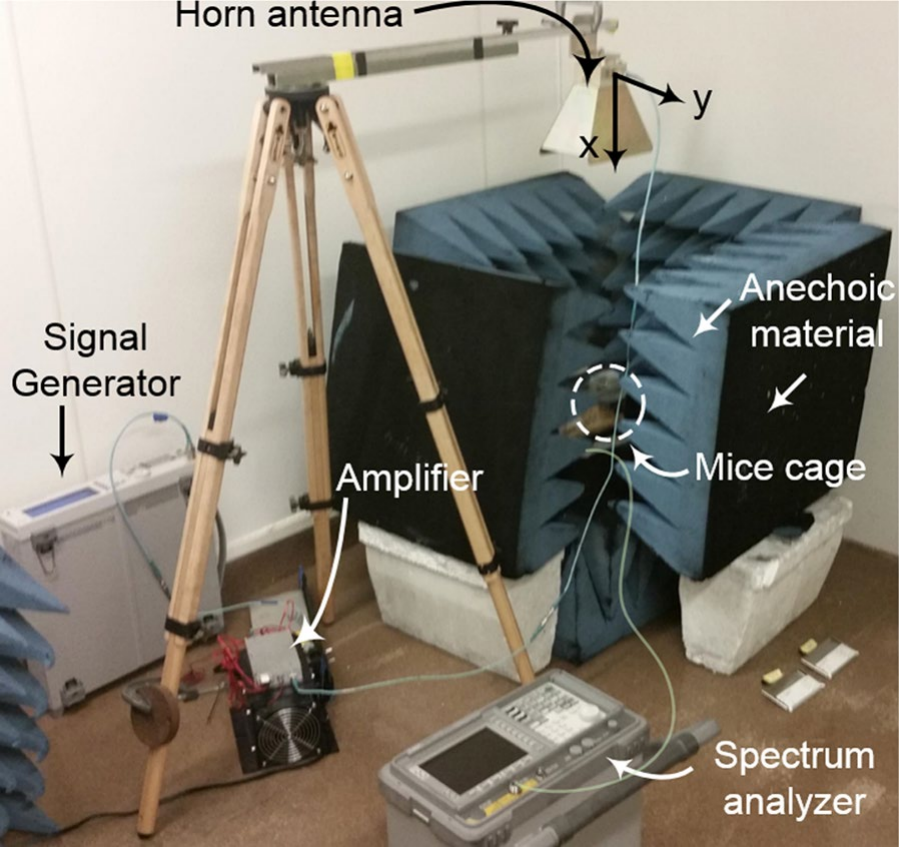
Photograph of the experimental setup for RF exposure of mice with all the apparatus used. Mice cage and patch is enclosed in the anechoic material but the horn antenna, spectrum analyzer, signal generator together with the amplifier can be seen. The horn antenna as setup is polarized in the z-direction

The uncertainty analysis of the test equipment was conducted on the equipment used to measure and record the power received by the treatment group. Performing uncertainty analysis on the equipment is an important step because it assists in ensuring that the power levels are below IEEE standard for continuous exposure and helps in comparing the results of RF experiments (IEEE Standard 1988). The equipment includes: a patch antenna, two transmission lines, and a spectrum analyzer. This setup can be seen in Fig. 2. With calibrated equipment, the assumption is that the uncertainty and loss data given by each respective data sheet is true. Table 2 shows the uncertainty values given for equipment used by the authors.

**Table 2:** Uncertainty of equipment

Equipment	Uncertainty
Biconical antenna	±1.00 dBm
Coax cables	±0.01 dBm
Spectrum analyzer	±0.40 dBm
Horn antenna	±2.00 dBm
Network analyzer	±1.30 dBm
Signal generator	±2.00 Hz

Other uncertainty values (i.e. patch antenna characteristics) were solved for by using the equations below. The power received at the patch antenna is:

1 $$PrdB = PRdB + 2 \times \left| {C_{l} dB} \right|$$

where *PrdB* is the power received at the patch antenna, *PRdB* is the power received at the spectrum analyzer, and *C*_*l*_ is the insertion loss of each transmission line. Because the transmission lines are calibrated we can make the assumption that

2 $$C_{l} dB = C_{l1} = C_{l2}$$

and the uncertainty of each transmission line is:

3 $$U_{{C_{l} }} = U_{{C_{l1} }} = U_{{C_{l2} }} .$$

Next, the power received at the patch antenna can then be converted into watts by:

4 $$Pr = 10^{{\frac{Prdb}{10}}} \, \left( {\text{W}} \right).$$

Then the power density incident on the antenna can then be calculated using Eq. 5 from the introduction:

5 $$S = \frac{Pr}{Ae}\,{\text{W}}/{\text{m}}^{ 2}$$

where S is the power density and *Ae* is the area of the effective aperture, computed by:

6 $$Ae = \frac{{Dr \times \lambda^{2} }}{4\pi }\,$$

*Dr* is the directivity of the patch antenna with an efficiency of 95 % and *λ* is the wavelength and defined by:

7 $$\lambda = \frac{c}{f}$$

where *c* is the speed of light in m/s and *f* is the frequency in hertz. The uncertainty of *λ* can be computed by

8 $$U_{\lambda } = \sqrt {\left( {\frac{\partial \lambda }{\partial f} \times U_{f} } \right)^{2} } .$$

Then the uncertainty of the effective aperture can be calculated as:

9 $$U_{Ae} = \pm \sqrt {\left( {\frac{\partial Ae}{\partial Gr} \times U_{Gr} } \right)^{2} + \left( {\frac{\partial Ae}{\partial \lambda } \times U_{\lambda } } \right)^{2} } .$$

Now the uncertainty of the power received at the spectrum analyzer can be computed. This value *U*_*PR*_ is dependent on the value recorded by the spectrum analyzer (PR) and its respective measurement tolerance (T_PR_) given by the manufacturer:

10 $$U_{PR} = \pm \frac{{\left| {10^{{\frac{{PR + T_{PR} }}{10}}} - 10^{{\frac{{PR - T_{PR} }}{10}}} } \right|}}{2}\,\left( {\text{W}} \right).$$

Knowing that the transmission lines are identical the equation can be simplified to:

11 $$U_{Pr} = \pm \sqrt {(U_{PR} )^{2} + 2 \times (U_{{C_{l} }} )^{2} } .$$

Knowing the uncertainty for the effective area of the aperture and the power received at the patch antenna. The uncertainty of the power density can be determined as:

12 $$U_{S} = \pm \sqrt {\left( {\frac{\partial S}{\partial Pr} \times U_{Pr} } \right)^{2} + \left( {\frac{\partial S}{\partial Ae} \times U_{Ae} } \right)^{2} }$$

**Fig. 3:**
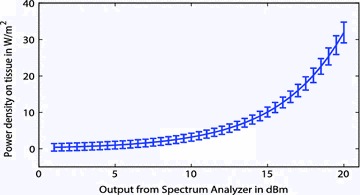
Uncertainty measurements for the equipment used to verify the level of RF power density exposure of the mice for a range of 1–20 dBm. This graph can be compared to the IEEE standard to ensure that mice are not being exposed to higher than allowable power density levels (IEEE Standard 1988)

**Fig. 4:**
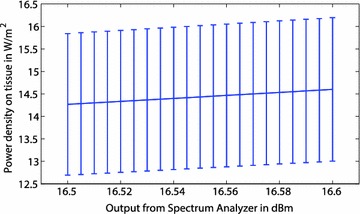
A close-up of the uncertainties for the range of power densities achieved when the spectrum analyzer reads in the range of 16.5–16.6 dBm. The maximum uncertainty value seen at 16.6 dBm must be less than the level recommended by IEEE for continuous exposure limits (IEEE Standard 1988)

These equations were used for the test equipment and the uncertainty of the power density was calculated. Figure 3 shows the relationship between the power levels of 1–20 dBm and their corresponding power densities. The error bars represent the uncertainty of the power densities. Figure 4 depicts the power levels of 16.5–16.6 dBm (power levels used by the authors) at increments of 0.005 dBm. Following the continuous RF energy exposure standards set by IEEE, and the uncertainty analysis provided here, power density levels can be set such that they will fall below the exposure maximum allowed (IEEE Standard 1988).

#### Uncertainty analysis of equipment used for characterization of the test room

**Fig. 5:**
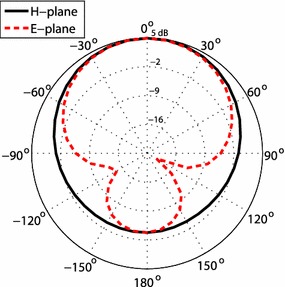
Measured radiation pattern in the principal xz-plane (*H-plane*) and yz-plane (*E-plane*)

The spectrum analyzer has a frequency range from 9 kHz to 3.0 GHz, and the biological antenna has a frequency range of 25 MHz–4 GHz (the setup is shown in Fig. 5). We assumed that the uncertainty values from the equipment data sheets were correct because the equipment was recently calibrated. Therefore, we calculate the power received by the spectrum analyzer as:

13 $$PrdB = PRba + 2 \times \left| {C_{l} dB} \right|$$

where *PrdB* is the power received at the spectrum analyzer, *PRba* is the power received at the biconical antenna. Knowing the uncertainty of both the biconcial antenna and the transmission lines, we can calculate the total uncertainty as follows:

14 $$U_{PrdB} = \pm \sqrt {\left( {\frac{\partial PrdB}{\partial PRba} \times U_{PRba} } \right)^{2} + \left( {\frac{\partial Pr}{{\partial C_{l1} }} \times U_{{C_{l1} }} } \right)^{2} + \left( {\frac{\partial Pr}{{\partial C_{l2} }} \times U_{{C_{l2} }} } \right)^{2} } .$$

Because the uncertainty of the transmission lines are equal, we can simplify this to:

15 $$U_{PrdB} = \pm \sqrt {(U_{PRba} )^{2} + 2 \times (U_{{C_{l} }} )^{2} } .$$

Using the uncertainty values provided for the characterization of the test room equipment, we are able to calculate the overall uncertainty of our equipment as approximately ±1.00 dBm.

#### Uncertainty analysis of equipment used for specific absorption rate experimental measurements

Incident power density and specific absorption rate (SAR) are commonly used to characterize RF energy exposure in the aforementioned exposure systems. Power density is the amount of power (in W) per unit area (in m). It can be calculated by

16 $$S = \frac{Pr}{Ae}\quad{\text{W}}/{\text{m}}^{ 2}$$

where S is the power density, Pr is the incident power at the aperture, and Ae is the effective area of the aperture.

SAR is a measure of electromagnetic (EM) energy absorbed by a body. SAR calculation can be accomplished through theoretical, experimental and empirical techniques. The Radio Frequency Radiation Dosimetry Handbook (Fifth Edition) 2009 describes both theoretical and experimental SAR techniques. A common theoretical SAR technique is electromagnetic (EM) simulation (e.g. COMSOL, HFSS, FDTD). Five common experimental SAR measurement techniques include:

1. Differential power measured in a closed exposure system.
2. Rate of temperature change in the biological test subject measured with noninterfering probes.
3. Calorimetric techniques.
4. Thermographic techniques.
5. Implantable E-field probes.

Finally, Durney et al. (1979) describes an empirical SAR technique. Which of these techniques are used to calculate SAR depend on the availability of resources. Using the equations found in Radio Frequency Radiation Dosimetry Handbook (Fifth Edition) 2009

17 $$P_{e} = P_{I} - P_{O} - P_{R} \quad \left( {\text{W}} \right) \,$$

and

18 $$P_{s} = P_{I} - P_{O} - P_{R} \quad \left( {\text{W}} \right) \,$$

where* P*_*e*_ is the power absorbed by the patch antenna in the empty enclosure;* P*_*I*_ is the input power;* P*_*O*_ is the output power;* P*_*R*_ is the reflected power; and* P*_*s*_ is the power absorbed by the patch antenna while the sample is present in the enclosure in Watts. After* P*_*e*_ and* P*_*s*_ are measured, the SAR is calculated by using

19 $$SAR = \frac{{\left| {P_{e} - P_{s} } \right|}}{{m_{(subject)} }} \quad \left( {{\text{W}}/{\text{kg}}} \right) \, .$$

Then the uncertainty for SAR is:

20 $$U_{SAR} = \pm \sqrt {\left( {\frac{dSAR}{dPe} \times U_{{P_{e} }} } \right)^{2} + \left( {\frac{dSAR}{dPs} \times U_{{P_{s} }} } \right)^{2} + \left( {\frac{dSAR}{dm} \times U_{m} } \right)^{2} } .$$

In Eq. 20, $U_{{P_{e} }}$ and $U_{{P_{s} }}$ are both equal to the uncertainty of power received at the spectrum analyzer, $U_{PrdB}$ in Eq. 14. Using these equations, the uncertainty of the SAR measurements was found to be 0.00034216 W/kg.

#### Microstrip patch antenna

**Fig. 6:**
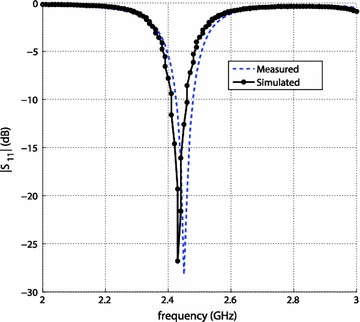
Reflection coefficient S_11_ values in dB

The microstrip patch antenna is widely used because of its low volume and thin profile characteristics (Balanis 2005). Also the microstrip antennas are inexpensive to manufacture using today’s modern printed-circuit technology and versatile in terms of resonant frequency, polarization, pattern and impedance. The microstrip patch antenna is a good candidate to be used as an antenna to measure the received power and calculate the power density for safe RF exposure of mice. Figure 6 shows the reflection coefficient S11 values for the simulated model (in HFSS) and measurements taken from a fabricated patch antenna.

##### Design and prototyping of rectangular microstrip patch antenna

**Fig. 7:**
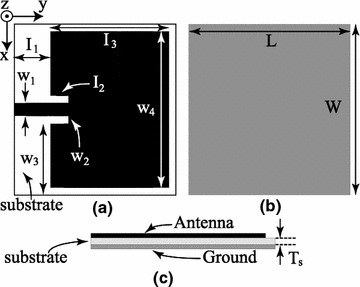
Geometry of the microstrip patch antenna. **a** Top view. **b** Bottom view and **c** side view. Structure characteristics: l_1_ = 11.8 mm, l_2_ = 2 mm, l_3_ = 27.5 mm, L = 50 mm, w_1_ = 2.1 mm, w_2_ = 1 mm, w_3_ = 20.85 mm, w4 = 45 mm, W = 48.5 mm and T_s_ = 1.52 mm

**Fig. 8:**
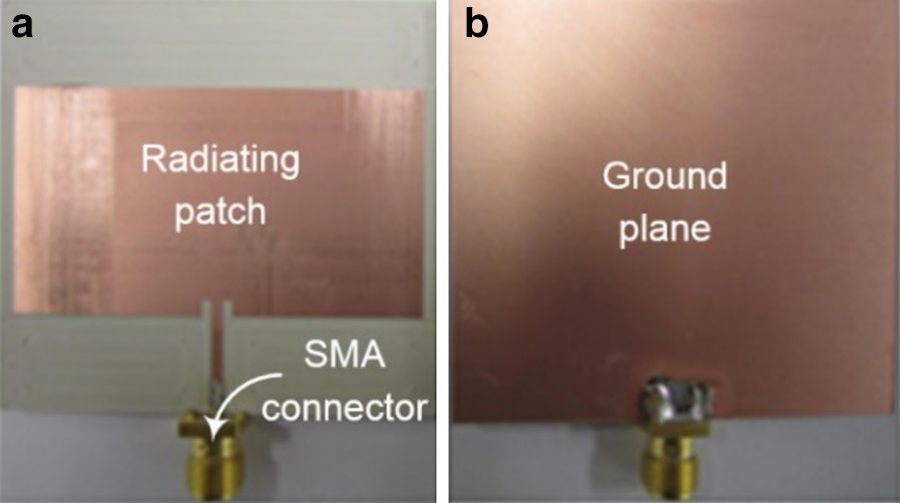
Fabricated sample of the patch antenna used for measuring the power density. **a** Top view. **b** Bottom view

A rectangular MPA with a microstrip feed is designed so its pattern maximum is normal to the top patch surface i.e., broadside radiator. Next, the actual length and width of the radiating patch are calculated using the design equations given in (Stutzman et al. 1998). The antenna is designed for the frequency of 2.43 GHz, which is in industrial, scientific and medical (ISM) radio bands. Also this is the same frequency that the mice will be exposed to using a horn antenna in a set of experiments conducted by the authors. The geometry of the microstrip patch antenna with detailed dimensions are shown in Fig. 7. The top layer is the radiating patch while the bottom layer is the ground plane of the antenna. As shown in the Fig. 7, the actual size of the radiating patch is 27.5 × 45 mm^2^ which is matched with 50 Ω using an inset-fed microstrip line. A detailed picture of the manufactured microstrip-fed rectangular patch antenna is shown in Fig. 8. To demonstrate the layout in Fig. 3, a prototype was designed using TMM4 (ɛ = 4.5, tan δ = 0.0020, copper thickness = 17.5 µm, and substrate thickness/Ts = 1.52 mm), manufactured and tested.

##### Simulation and measured results of the MPA

All the properties of a MPA mentioned in the previous section were used in the full wave design tool, Ansoft HFSS (Balanis 2005), to simulate and optimize the results prior to fabrication. The simulated and measured results of the reflection coefficient are shown in the Fig. 8, which shows good agreement between the simulated and measured results. A slight shift in the resonance frequency is due to the fabrication tolerance. Also these results show that the antenna is matched with a 50 Ω port. Furthermore, Fig. 5 shows the measured radiation pattern of the MPA which is broadside. The pattern shows high back radiation which is due to the small ground plane. Small ground plane was used because of good impedance matching at the resonant frequency. The antenna is linearly polarized in y-axis according to the orientation used in the Fig. 7. The gain of the antenna was measured using gain comparison method and found to be 4.8 dBi.

### Characterization of the test room

Before the experiment began, the radio frequency energy profile of the room was characterized on the x, y, and z-axes. This characterization process is to determine if any unwanted RF energy is present in both the test area and the control area of the mouse room. In order to characterize the RF energy, a spectrum analyzer is attached to a biconical antenna. The connection is made with two transmission lines, each 61 cm long connected via Agilent interconnects.

**Fig. 9:**
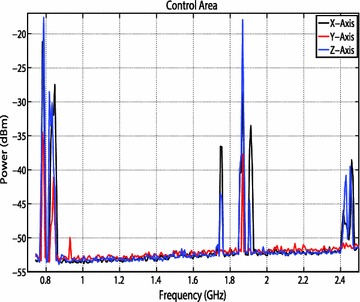
Maximum RF values for the control area at frequencies of 700 kHz to 2.5 GHz for the control area over the 24 h study

**Fig. 10:**
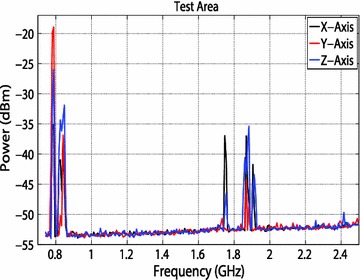
Maximum RF values for the test area at frequencies of 700 kHz to 2.5 GHz for the test area over the 24 h study

The biconical antenna is placed on a wooden tripod approximately 122 cm tall and placed in the area of the room where the control mice would reside throughout the study. The direction of the antenna coincided to the x-axis. Photographs were taken of the setup noting the position and direction of the antenna. Figure 5 shows the antenna polarized in the z-direction. The spectrum analyzer saved the highest recorded power using the ‘Hold Maximum’ function for each frequency during the duration of the characterization process. Data were recorded for 24 h. The experiment was then repeated, by moving the antenna to be polarized in the y- and x- axes. Finally, the process was repeated in the area where the test mice would reside throughout the RF exposure study. Trends in the RF energy can be seen at various frequencies in Figs. 9, 10. The spikes are most likely caused by various electronic devices. For example, WiFi has a frequency of 2.4 GHz, and cellphone providers commonly use 0.7–0.9 and 1.9 GHz bands. The change in RF energy can be caused by a wide variety of factors. For example, spikes are much more prevalent for the x-axis for the control area, most likely because the biconical antenna was pointed toward the hallway.

The increased traffic in the hallway easily could have contributed to the increased RF activity and spikes in the graph. In addition, the high spikes seen for the Z-axis for the control area could be attributed to the ductwork directly above the antenna. All values recorded during the 24 h time period were maximum values, meaning there is no way to know how long the mice were exposed to these levels of RF energy. These energy spikes could have occurred sporadically throughout the test, or they could have remained fairly constant. However, most spikes did not exceed more than −20 dBm, which is well below the exposure power level of the treatment group. Nonetheless, it is important to limit as much unwanted RF energy as possible. Therefore, anechoic material was set up around both the test and the control mice to limit extraneous RF energy exposure. In addition, during the experiment, cellphones and other electronic devices were not allowed in the test room.

#### Experimental specific absorption rate calculation

**Fig. 11:**
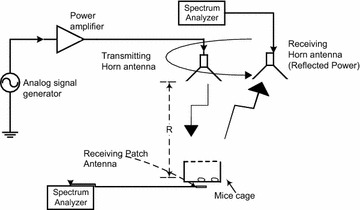
Shows the SAR experimental setup. Using a horn antenna to transmit a power density of 1.6 mW/cm^2^, and a patch antenna to measure the incident power. A horn antenna was used to measure the reflected power, this was conducted over eight locations ~38 cm away from the transmitting horn antenna

For this study the SAR was calculated using the differential-power technique and empirical calculations. Figure 11 shows the experimental setup for the SAR calculations. In short, the procedure was conducted inside an anechoic chamber to remove any outside EM noise and provide a well-defined environment. A horn antenna connected to a power amplifier and signal generator transmitted an EM field at 2.45 GHz with a maximum power density of 1.6 mW/cm^2^. This power density was measured by a patch antenna located below a plastic cage and recorded by a spectrum analyzer (in dBm). A horn antenna was used to measure the reflected power at eight different locations in a circular plane with a radius of 38.0 cm at the level of the transmit antenna (as shown in Fig. 11) and recorded by a spectrum analyzer (in dBm). The average whole body SAR was measured to be 0.3422 ± 0.00034 W/kg at a maximum power density of 1.6 mW/cm^2^ which compares well to empirical SAR calculations using the equations found in Durney et al. (1979), which calculates a value of 0.3750 W/kg for small animals.

#### Example RF exposure system implementation

Following calibration of equipment, and characterization of background, mouse cages are adopted for RF studies. This involved using non-metal cages, food trays, and water dispensers. In addition, a Plexiglas top is added with many ventilation holes (~0.635 cm.) to ensure that mice remain in their respective cages. For radio-frequency exposure experiments BALB/c mice (6–9 weeks of age) were obtained from Jackson laboratory (Bar Harbor, ME, USA). Animals were housed on Alpha-dri™ paper bedding (Shepherd Speciality Papers, Watertown, TN, USA) in micro filter-topped cages (Ancare, Bellmore, NY, USA) in a specific pathogen-free facility with ad libitum access to food and water.

The mice are then separated into treatment and control groups respectively. Anechoic material is used in both the treatment and control group to limit the exposure of background RF and to ensure that the control group is not radiated with stray test RF energy. Figure 2 shows the equipment setup for the treatment group. According to the reference system used in Fig. 2, the horn antenna is polarized in z-direction and receiver antenna was placed in the same direction in which horn is polarized. Moreover, this distance ‘R’ is crucial for receiving safe power density at the right level i.e., top of mice body as shown in Fig. 2. This distance is dependent on the frequency used, gain (G_t_) of the Horn antenna and power transmitted (P_t_) from the Horn antenna. Equation 21 shows that relationship:

21 $$\Pr \, \left( {dBm} \right) = Pt \, \left( {dBm} \right) + Gt \, \left( {dB} \right) + Gr \, \left( {dB} \right).$$

This relationship is known as Friis’s transmission equation (IEEE Standard 1988). In order to ensure that the treatment mice are receiving the correct dose of RF energy, a patch antenna connected to a spectrum analyzer was used to record the power received. This power received by the mice is below the standards set by IEEE which, for example, is 1.6 mW/cm^2^ for 2.45 GHz (IEEE Standard 1988). Using uncertainty analysis, RF power density levels are set and the power level within the anechoic material was also mapped to verify that the power-density levels were below IEEE safe exposure standards. Mice were placed within their control or treatment cages. Treatment mice can then be exposed to RF energy for a set duration.

## Discussion

This experimental exposure system can be used for repeatable long term far-field RF exposure for freely-moving mice. The equipment used promotes convenient uncertainty analysis that in turn provides more accurate power density and SAR estimates. In addition, anechoic material reduces potential environmental effects on these estimates and the steps outlined in this work can be easily changed to include many different experimental parameters (e.g. frequency, time of exposure, signal type, pulsed or continuous).

An improvement to the exposure system reported here includes an independent estimate of SAR based on full-wave electromagnetic simulations and theoretical computations on a 3D whole mouse model. This would allow for independent verification of the experimental differential power procedure used here to estimate SAR and found in the Radio Frequency Radiation Dosimetry Handbook (Fifth Edition) 2009.

## Conclusion

This manuscript describes a new far field RF exposure system for unrestrained murine models that reduces experimental error. The steps to reduce experimental error were described and the result of this manuscript is an experimental exposure system to be applied to future biological studies.

## Abbreviations

RF: radio-frequency
WHO: World Health Organization
IEEE: Institute of Electrical and Electronics Engineers
SAR: specific absorption rate
